# A Direction-independent, High-density Mapping Catheter Provides Electrophysiological Advantage in Complex Atrial Tachycardia Ablation Following Pulmonary Vein Isolation

**DOI:** 10.19102/icrm.2021.121203

**Published:** 2021-12-15

**Authors:** Gregory Woo, Catherine Markert, Rebekah Montgomery

**Affiliations:** ^1^Cardiovascular Services, Department of Medicine, CaroMont Regional Medical Center, Gastonia, NC, USA; ^2^Abbott, Chicago, IL, USA

**Keywords:** Cardiac mapping, high density, HD Grid, wavefront

## Abstract

Catheter ablation of recurrent atrial arrhythmias following pulmonary vein isolation can be challenging given the complex nature of previously ablated tissue, and managing these already complex cases may be rendered more difficult by the impact of wavefront directionality on mapping catheter orientation, which can make the accurate identification of arrhythmogenic substrate more difficult to achieve. In this report, a 72-year-old man with a history of symptomatic paroxysmal atrial fibrillation and prior pulmonary vein isolation (PVI) underwent repeat ablation. Importantly, this case study demonstrates how a direction-independent high-density mapping catheter (Advisor™ HD Grid; Abbott, Chicago, IL, USA) can identify fractionated low-voltage zones that may be missed when using a standard linear ablation catheter.

## Case presentation

Catheter ablation of recurrent atrial arrhythmias post–pulmonary vein isolation (PVI) can be challenging due to the complex nature of previously ablated tissue.^1^ The challenges of these already complex cases can be compounded by the impact of wavefront directionality on mapping catheter orientation, thus complicating the accurate identification of arrhythmogenic substrate.^2^ This case study demonstrates how a direction-independent high-density mapping catheter (Advisor™ HD Grid; Abbott, Chicago, IL, USA) can identify fractionated low-voltage zones that may be missed using a standard linear ablation catheter.

A 72-year-old man with a history of symptomatic paroxysmal atrial fibrillation, and a prior PVI performed with a 28-mm cryoballoon catheter (Arctic Front™; Medtronic, Minneapolis, MN, USA), presented one year after the index PVI with recurrence of palpations. Electrocardiography was performed, which demonstrated an atrial tachycardia (AT) with 1:1 conduction.

**Figure 1:**
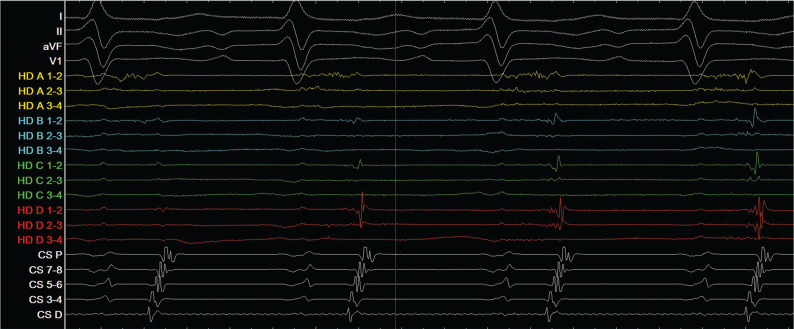
Sweep speed: 200 mm/s. AT with a cycle length of 490 ms. HD A 1-2 demonstrates a high-frequency fractionated signal. The onset portion of the signal is 90 ms earlier than the P-wave. CS: coronary sinus.

The patient opted for repeat electrophysiology study and ablation. The procedure was performed under general anesthesia. Catheters were positioned along the tricuspid annulus, His-bundle region, and coronary sinus (CS). The patient presented in tachycardia with a cycle length of 490 ms and eccentric CS catheter activation with earliest activation in the distal CS, indicating a left atrial origin **(Figure 1)**. Left atrial access was obtained via a transseptal puncture, and electroanatomic mapping (EnSite Precision™; Abbott, Chicago, IL, USA) was performed with a multi-electrode grid catheter.

**Figure 2:**
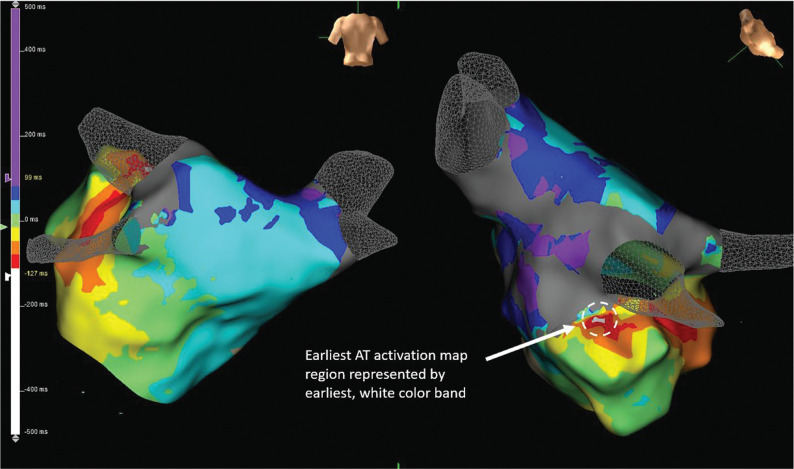
Activation map. Focal AT appears to be originating from the superior ostium of the left atrial appendage. AT: atrial tachycardia.

Activation and voltage mapping of the 490-ms tachycardia were simultaneously performed.^3^ Dense scar zones were defined as regions where the local signal was less than 0.07 mV in amplitude. Both bipolar (30-Hz high-pass, 300-Hz low-pass, notch filter) and unipolar (0.5-Hz high-pass, 100-Hz low-pass, notch filter) filters were used to assess the left atrial signals and identify potential ablation targets. Entrainment mapping was performed on both the left atrial roof and mitral isthmus areas, indicating that those regions were not critical to the tachycardia. Activation mapping suggested a focal AT originating from the superior ostium of the left atrial appendage **(Figure 2)**. A low-voltage, fractionated signal was identified with the distal component 90 ms prior to the onset of the P-wave **(Figure 1)**. The location was marked on the three-dimensional map.

**Figure 3:**
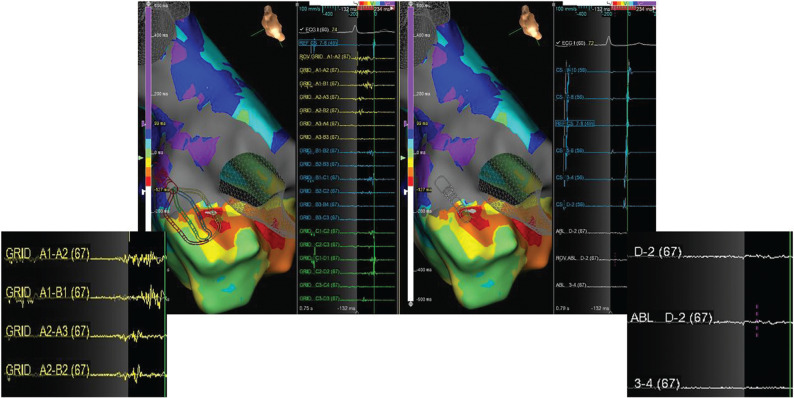
**Left:** Grid catheter at the successful site of ablation in the superior ostium of the left atrial appendage. A high-frequency, fractionated signal is readily identifiable on the A spline. **Right:** Ablation catheter also positioned at the successful site. Signal amplitude is diminished, and fractionation is difficult to identify.

The grid mapping catheter was exchanged for an externally irrigated ablation catheter (FlexAbility; Abbott), which was positioned at the suggested focal origin. Ablation catheter–tissue contact was confirmed using intracardiac echo and impedance. Neither the bipolar nor the unipolar atrial electrograms (EGMs) on the ablation catheter were able to be appreciated as they had appeared on the grid catheter, which produces EGMs independent of the electrode orientation to wavefront activation **(Figure 3)**. Radiofrequency (RF) energy was delivered at a power of 35 W. Within three seconds of RF application, the tachycardia terminated.

The tachycardia was not re-inducible with burst atrial pacing down to 200 ms. At the time of the patient’s six-month follow-up, he remained arrhythmia-free.

This is a case study and therefore did not undergo the institutional review board process.

## Discussion

The incidence of ATs after cryoballoon ablation has been reported to be as high as 8%.^4^ ATs can be focal or re-entrant, and are caused by one or a combination of triggered activity, enhanced automaticity, or reentrant activity. Furthermore, it has been shown that the substrate required for an AT may be correlated to areas of abnormal myocardium, such as those with fibrosis, hypertrophy, and thinning.^5^ Fibrotic tissues produce signals which are difficult to discern, thereby increasing the level of complexity associated with mapping an AT.^3^

Due to its grid design, the grid mapping catheter can assess the timing and voltage in a direction-independent manner, thereby reducing one of the challenges associated with these complex procedures. The catheter features a rectangular-shaped design with 16 1-mm electrodes that are equally spaced along the splines and across the splines (3 mm edge to edge). The electrical signals recorded between the orthogonal pairs are compared, and the highest amplitude signal is selected for the annotation of the localized timing (using EnSite Precision™; Abbott). This catheter design and algorithm allows for the creation of voltage and activation maps that are more accurate due to the reduction of “bipolar blindness”^2^—a phenomenon that occurs when the electrodes’ angle of incidence to the myocardium records near-simultaneous activation on both poles, thus canceling out most of the bipolar signal.^6^

Our case demonstrates the contrast in appreciable signals between a grid catheter and a linear 3.5-mm tip ablation catheter. When the ablation catheter was placed at the successful site of termination, which had been identified by the grid catheter as early and fractionated, the earliest AT signal was not able to be readily identified **(Figure 3)**. This is potentially due to a combination of three variables that affect the resultant bipolar EGMs: electrode size, spacing, and incident angle.^7^ First, the 3.5-mm distal electrode tip of the ablation catheter creates a large antenna, thereby acquiring a signal that is representative of a larger swath of tissue than that which is in contact with the ablator.^6^ Second, the closest electrode pair to the tissue surface, excluding the large distal 3.5-mm antenna, is spaced 4 mm apart on the ablator. In contrast, the grid catheter’s electrodes have a 3-mm spacing and are able to acquire a more near-field signal.^6^ Third, the ablation catheter’s linear design, and thus the singly oriented point of view, does not allow for a direction-independent analysis of wavefront propagation.^6^ In an exposition of the current state of substrate-based ablation, Josephson et al. recommend standardized catheters with small electrodes and minimal interelectrode distance for minimizing poor resolution issues associated with recording techniques.^6^ These recommendations, combined with the elimination of incident angle inaccuracy, allowed the grid catheter to create a detailed activation map of the tachycardia, allowing the authors to determine the tachycardia mechanism and identify the successful termination site.

## Conclusion

The difference in appreciation between complex signals on a linear 3.5-mm tip ablation catheter and a grid-style high-density mapping catheter can be substantial. The inability of an ablation catheter to visualize one such complex signal does not negate the site’s viability for successful ablation. This case highlights the importance of collecting and analyzing complex circuits using a high-density, direction-independent mapping catheter.
